# Internal and external risk factors analysis on pilot precondition in Indonesia

**DOI:** 10.12688/f1000research.163883.1

**Published:** 2025-04-24

**Authors:** Inne Yuliawati, Budi Sampurna, Tjhin Wiguna, Imam Subekti, Aria Kekalih, Widura Imam Mustopo, Hervita Diatri, Wawan Mulyawan

**Affiliations:** 1 Medical Science, Doctoral study program, Universitas Indonesia, Jakarta Pusat, Jakarta, Indonesia; 2 DGCA, Ministry of Transportation, Indonesia, Directorate General of Civil Aviation, Jakarta Pusat, Indonesia, Indonesia; 3 Community Medicine, Aerospace Medicine Study Program, Universitas Indonesia, Jakarta Pusat, Jakarta, Indonesia; 4 Ethic and Medicolegal, Rumah Sakit Dr Cipto Mangunkusumo, Central Jakarta, Jakarta, Indonesia; 5 Psychiatry, Universitas Indonesia, Jakarta Pusat, Jakarta, Indonesia; 6 Psychiatry, Child and Adolescent Psychiatrist, Rumah Sakit Dr Cipto Mangunkusumo, Central Jakarta, Jakarta, Indonesia; 7 Internal medicine, Universitas Indonesia, Jakarta Pusat, Jakarta, Indonesia; 8 Internal medicine, Division of Endocrinology and Metabolism, Rumah Sakit Dr Cipto Mangunkusumo, Central Jakarta, Jakarta, Indonesia; 9 Occupational Medicine, Universitas Indonesia, Jakarta Pusat, Jakarta, Indonesia; 10 Psychology, Universitas Jayabaya, East Jakarta, Special Capital Region of Jakarta, Indonesia; 11 Aviation, National Transport Safety Committee (NTSC) of the Republic of Indonesia, Central Jakarta, Jakarta, Indonesia; 12 Patient Safety, Rumah Sakit Dr Cipto Mangunkusumo, Central Jakarta, Jakarta, Indonesia

**Keywords:** pilot, error, precondition, internal and external factors, HFACS

## Abstract

**Background:**

Pilot errors accounted for 80% of accidents and 50% of serious incidents. The Human Factor Analysis and Classification System (HFACS) allows the identification of pilot preconditions as an imminent layer of error. This study aimed to analyze the correlation between internal and external risk factors and pilot preconditions in Indonesia.

**Methods:**

A cross-sectional study design with purposive sampling, directed to male pilots who had flight duty in the past seven days, underwent medical examination at the Aviation Medical Center, Jakarta, August 12–16, 2024. The data were collected through a self-report questionnaire, Trail Making Test A and B, laboratory tests (plasma lipid, fasting blood glucose), and physical measurements (height, weight, waist circumference, and blood pressure). The independent variables included internal (Age, Burnout, Metabolic Syndrome parameters) and external (Flight Time, Duty Time, Unscheduled Flight Duty, Number of Sectors, Sleep Duration). The dependent variables were pilot preconditions based on the HFACS.

**Results:**

A total of 122 participants participated in this study. Among them, 28.7% had an unscheduled flight duty in the last 30 d. Significant correlations were found between Adverse Mental State and HDL-cholesterol (95%CI=1.52 – 5.80), Adverse Physiological State and Burnout [personal (95%CI=0.005–0,04), work-related (95%CI=0.009–0.042)], Physical Mental Limitation and Fasting Blood Glucose (95%CI =(-0.479)–(-0.071)), and Number of Sectors (95%CI=0.022–3.001). For Personal Readiness (PR), a significant correlation was found between PR-psychological demand and Flight-Time One Year (95%CI=(0.000 – 0.001), Sleep Duration (95%CI =(-0.137)–(-0.013)), Waist Circumference (95%CI =(-0.014)–(-0.002)), PR-Social Support and Sleep Duration (95%IK=0.018–0.207), and Client-Related Burnout (95%IK =(-0.011)–(-0.002)).

**Conclusions:**

The internal factors that correlated with pilot preconditions in Indonesia were waist circumference, high-density lipoprotein cholesterol, fasting blood glucose, personal burnout, work, and client-related burnout. External factors that correlated with pilot preconditions were the number of sectors, Flight-Time One Year and Sleep duration. These findings emphasize the need to address both physical and mental health aspects of pilots to enhance aviation safety.

## Background

Several studies on human factors have shown that human errors accounts for 70-80% of aviation accidents and serious incidents. ^1– 4^ Identifying human errors that compromise aviation safety is crucial to mitigate aviation accidents and serious incidents. To understand human error, it is pivotal to identify the factors contributing to human errors, particularly human factors. Human factors are complex and consist of an interaction of physiological, psychological, and psychosocial aspects that comprehensively affect cognitive function. Thus, to identify human error, researchers have developed human error and human factor models. One of the models that has been widely implemented is the ^1^Human Factor Analysis and Classification System (HFACS). ^5, 6^

The HFACS is a structured and practical tool for classifying human factors that contribute to accidents and serious incidents in aviation. The HFACS allows the identification of multiple contributory factors that generate failure in aviation accidents or incidents, not only by enumerating human errors, but also by evaluating forward to the higher cognitive process, which is the precondition, then highlighting the supervisory deficiencies and the organizational factors. ^4– 6^ Li et al. (2006) stated that safety commitment should start from the highest level, which is the organizational influence, then the commitment progresses linearly to the next level, unsafe supervision, precondition of unsafe acts, and the unsafe acts of the operator. ^6^

According to HFACS, the highest level of organizational influence consists of resource management, organizational climate, and operational processes. The Third layer, or Unsafe Supervision, comprises inadequate supervision, planned inappropriate operation, failed to correct problems, and supervisory violation. The next layer or The second layer is the Precondition of Unsafe Acts that encompasses the Physical Environment, Technological Environment, Adverse Mental State, Adverse Physiological State, Physical Mental Limitation, Crew Resource Management and Personal Readiness. The last or first layer is an unsafe act that involves Errors and Violation. Errors include decision errors, skill-based errors, and perceptual errors. Violation comprises routine and exceptional violations. ^6^

Because the precondition is the imminent layer prior to human error and pilots are the key element to aviation safety, it is critical to heed the precondition of pilots whose cognitive function is influential in managing the precautionary measures of errors. Li et al. (2001) emphasized that pilot errors account for 80% of accidents and 50% of serious incidents in the United States of America. ^6– 8^ In addition, and based on accident and serious incident data from 1983 to 1996, Li et al. (2001) stated that pilot errors account for 89% of commercial aircraft accidents and 94% of charter and commuter aircraft accidents. Thus, humans, specifically pilots, are the last resource for aviation safety, and it is important to mitigate the risk that pilots have as preconditions. ^8^

The preconditions of pilots based on HFACS Level 2 are as follows: adverse mental health, adverse physiological state, physical mental limitation, cockpit resource management, and personal readiness. An Adverse Mental State is described as one or more of these conditions, including stress, loss of situational awareness, mental fatigue/burnout, distraction, and channelized attention *.* ^9– 11^ In terms of Adverse Mental State, stress and burnout are mental health problems that could cause depression. Depression is a major mental health problem that can jeopardize aviation safety because it progressively causes inflight incapacitation and suicide. Ackland et al. (2022) stated that at least one pilot uses an aircraft for suicide every year and contributes to 3.75% of all incapacitations that cause accidents. ^12^

The Adverse Physiological State is described as one or more of these conditions, including fatigue, hypoxia, spatial disorientation, and any medical condition. ^9^ Fatigue in aviation is a major contributing factor to accidents, and several studies have been conducted to investigate the risk factors of fatigue in aviation. In addition, a decrease in medical conditions requires an attentive approach to prevent inflight incapacitation in pilots. In terms of medical condition, with sedentary lifestyle and the concentration of high fat, sodium and sugar in processed food consumption, metabolic syndrome is a major health concern, not only in Indonesia but also worldwide. The National Transport Safety Committee (NTSC) ^13^ reported in the investigation report that a pilot with a history of obesity and dyslipidemia most likely experienced daytime sleepiness while flying, crashing the aircraft on April 12 ^th^ 2017, in Oksibil, Papua, Indonesia. ^14^

The Cockpit Resource Management (CRM) is described as one or more of these conditions including lack of communication, power distance/authority, lack of teamwork, lack of pre-flight briefing, lack of leadership. ^9^ CRM is an interpersonal interaction that consists of crew coordination, cockpit management and personal capability. CRM contributed significantly to inflight safety. The NTSC reported that the lack of crew coordination that caused poor communication and steep authority had been a cause for the accident on May 17 ^th^ 2011, in Kaimana, Papua. ^15^

Physical Mental Limitation is described as one or more of these conditions, including limitations in motoric movement or sensory input and insufficient reaction. ^9^ Cognitive function is responsible for escorting the accurate reaction in a timely manner for pilots, because every second in aviation is significant in creating flight safety. Cognitive function receives sensory input and manages critical thinking to create significant reactions by engaging in motor movements. Gaur et al. (2005) stated that 31,3% of accidents in India accounted for physical and mental limitations. ^5^

Lastly, the precondition of unsafe acts is Personal Readiness, which is described as one or more of these conditions, including sleep restriction, lack of experience/competency, unscheduled flight duty, drinking alcohol, lack of information, and injury/decrease in medical fitness. ^9^ Unscheduled flight duty could cause sleep restriction and fatigue that compromises flight safety. Thus, it is important to investigate the risk factors for the preconditions of pilots and ultimately mitigate pilot errors.

In terms of pilot preconditions, the risk factor for pilot error is divided into two factors: internal and external. Internal factors are physiological and psychological factors related to pilots, such as age, medical condition (mental fatigue or burnout, blood pressure, lipid profile, blood glucose, and waist circumference), and alcohol drinking habits. External factors include physio-psychosocial factors or work-related factors such as flight time, flight duty time, number of sectors, unscheduled flight duty, and sleep duration).

Ultimately, Aviation safety, specifically pilot errors and preconditions, are a major concern in Indonesia. Indonesia is an archipelagic country that is geographically strategic, allowing it to support 201 airports (33 of which are international airports), 61 commercial airlines, and 30 charter and commuter airlines. In addition, connectivity by air transport is highly active and important for its economic growth. ^16^ For example, at Soekarno-Hatta International Airport in Cengkareng, the average number of aircraft activities is 80 aircraft movements per hour. ^17^ Additionally, the NTSC recorded that human factors accounted for 66 out of 69 accidents and serious incident investigation reports from 2014 to 2023 in Indonesia. Thus, this condition requires attentive measures regarding human factors, specifically the pilot’s risk factors of precondition in Indonesia.

This pilot study investigated pilots’ risk factors for preconditions based on HFACS in Indonesia. This study aimed to investigate the correlations between internal and external risk factors and preconditions among pilots in Indonesia.

## Methods

### Procedures

The study was approved by the Medical Research Ethics Committee, Faculty of Medicine, University of Indonesia (certificate number KET1139/UN2). F1/ETIK/PPM.00.02/2024, dated 5 August, 2024. The Sample Size was determined with sample size for correlation analysis, using Open Epi Sample Size 2.0, with coefficient correlation (r) = 0,3; The standard normal deviate for α = 0.05; Z _α_ = 1,96 and The standard normal deviate for β = 0,2 Z _β_ = 0,842. The 10% corrected sample size included 117 participants.

This was a cross-sectional study, and data were collected using purposive sampling. The participants were adult pilots who had their medical examinations at Balai Kesehatan Penerbangan (Aviation Medical Centre), Directorate General of Civil Aviation in Jakarta, August 12–16, 2024. The written informed consent were obtained by giving a thorough explanation of the procedure and ethic of the study, suggesting each participant to read the explanation and ask questions related to their participation. Data were collected from the participants who gave their consent to participate in the study, by filling in the anonymous self-report questionnaires and taking the Trail Making Tests A and B, while the laboratory test results, including plasma lipid and glucose levels, and physical examination results including height, weight, waist circumference, and blood pressure, were collected from the participants’ medical records. The inclusion criteria were male pilots who were actively flying for flight duty in the last seven days. Because the physiology of metabolism and hormonal regulation differs between genders and there are only two active female pilots in Indonesia, the participants were not directed to female pilots. In addition, during data collection, there were no female pilots undergoing medical examinations.

The independent variables were divided into external and internal variables. The external variables were pilots’ workload-related factors, ^18– 21^ including Flight Time in the last 24 consecutive hours, in the last seven days, in the last 30 days, and in the last one year, and Total Flight Time; Flight Duty Time; Sleep Duration; and Unscheduled Flight Duty in the last 30 days. The internal variables were age (in years), alcohol drinking habits, burnout, and other internal factors that were collected from participants’ medical records were waist circumference (in cm), systolic blood pressure (in mmHg), diastolic blood pressure (in mmHg), Fasting Blood Glucose (FBG, in mg/dL), triglyceride (mg/dL), and high-density lipoprotein (HDL) cholesterol (in mg/dL).

Alcohol drinking habits were measured using the Cut-down, Annoyed, Guilty, Eye-opener (CAGE) questionnaire ^22^ ( https://portal.ct.gov/-/media/dph/maternal-mortality/cage-substance-screening-tool.pdf) by giving a 0 scale for a No answer and a 1 scale for a Yes answer. The possible total score range for all scales is 0 to 4. The total score was the sum of each value on the scale.

Burnout was measured using the Copenhagen Burnout Inventory (CBI) questionnaire consisting of three dimensions, ^23, 24^ with a total of 19 questions. The first dimension was personal burnout, consisting of six questions; work-related burnout, consisting of seven questions; and client-related burnout, consisting of six questions. All questions were answered using a Likert scale, ranging from 0 (almost never/to a very low degree), 25 (seldom/to a low degree), 50 (sometimes/somewhat), 75 (often/to a high degree), and 100 (always/to a very high degree). Exclusively for question 13, the scale is inversed, where the 0 value indicates always or to a very high degree, 25 being often or to a high degree, and so on. The questionnaire had a reliability value of Cronbach Alpha of 0.94 ( https://www.researchgate.net/publication/247511197_The_Copenhagen_Burnout_Inventory_A_new_tool_for_the_assessment_of_burnout ). The possible score range for all scales was 0–100. Scores for each subscale were averaged, and the total averaged score was calculated.

The dependent variables were the preconditions of pilots based on level two HFACS, the precondition of unsafe acts, consisting of five variables: Adverse Mental State, Adverse Physiological State, Physical Mental Limitation, Crew Resource Management, and Personal Readiness. First, the Adverse Mental State was measured using the Holmes Rahe Questionnaire, ^25, 26^ which consists of 43 questions about life events that may occur in the participant’s last year. Each life event in the questionnaire was assigned a value of. The final score of the questionnaire is the sum of each life event’s assigned values multiplied by the frequency with which each event has happened in the participant’s last year. The questionnaire has a Cronbach Alpha 0.96-0.86 (DOI: 10.1093/occmed/kqx099).

The Adverse Physiological State measured by the Fatigue Severity Scale questionnaire ^27– 29^ consists of nine questions that represent the participants’ perception of their fatigue in the last 7 days (DOI: 10.1007/978-1-4419-9893-4_35). The questionnaire was assessed using a Likert scale ranging from 1 to 7, where the higher the value of the scale, the higher the level of fatigue. The final score on the questionnaire was the average value of the answers to each question.

A participant’s Physical Mental Limitation was identified using the Trail Making Test (TMT) A and B, ^30, 31^ which represent the participant’s cognitive function. The duration (in seconds) to complete TMT A and B of each participant was recorded, and then the physical mental limitation was measured by the absolute value of the difference between the time participants took to complete TMT A and B.

Cockpit Resource Management was measured using the Cockpit Management Assessment Questionnaire (CMAQ), ^32^ which consisted of 25 questions. Each question is measured using a Likert scale such that it is possible to quantify the behavior in crew coordination, cockpit management, and personal capability while fatigued and stressed. The Likert scale implied in the questionnaire ranges as 1 (strongly agree), 2 (disagree), 3 (neutral), 4 (agree), 5 (strongly disagree). The output of the questionnaire was obtained from the average of the Likert-scale values answered for each question. The questionnaire had an internal consistency Cronbach alpha value of 0.74-0.81 (DOI: 10.1037/0021-9010.75.6.682).

Personal Readiness was measured by the Job Content Questionnaire (JCQ). ^33^ JCQ measures four dimensions of personal readiness, with a total of 34 questions. Each dimension followed by question amount was psychological demand with nine questions, decision latitude with 12 questions, skill discretion with five questions, and social support at work with eight questions. The answers to each question were measured with a Likert scale ranging from 1 to 4, where the higher the value, the more the participant agrees with each question on the questionnaire. The final data from the questionnaire included the average scores obtained for each dimension. The questionnaire has a reliability value of Cronbach Alpha 0.71-0.86 (DOI: 10.1037//1076-8998.3.4.322).

### Statistical analysis

Statistical analyses were performed using IBM Statistical Package for the Social Sciences (SPSS) version 22 software. Descriptive statistics for each variable were used to assess prevalence. All the variables were numeric, with the analysis starting from descriptive analysis of the mean or median. Bivariate analysis was performed with a parametric test (Pearson if the data distribution was normal; Spearman if the data distribution was not normal). Analysis was performed to assess the correlation between the dependent and independent variables. Statistical significance was set at p-value of <0.05. Subsequently, multivariate analysis with linear regression was performed to analyze variables with a p-value of <0.2.

## Results

### Characteristic

A total of 122 male pilot subjects were collected, 79.7% of which were active pilots flying for commercial airlines or operating on an Airworthiness Operation Certificate (AOC) 121, 14.8% were active pilots flying for charters and commuters or operating on AOC 135, and 3.3% were active pilots flying for general aviation or operating on AOC 91. In terms of unscheduled flight duty, 28.7% of them had unscheduled flight duty in the last 30 days, with a frequency ranging from to 1-3 and a median of 2 times, while for alcohol drinking habit, 18.8% of them had a habit of drinking alcohol with CAGE scores ranging from 0 to 4. The median age of the subjects was 33 years (range: 19–61 years). The mean waist circumference among the subjects was 92.96 cm (Standard Deviation/SD=12.90), Systolic Blood Pressure was 126.92 (SD=10,39) mmHg, Diastolic Blood Pressure 79.08 (SD=5.63) mmHg, Fasting Blood Glucose 101.77 (SD=13.58) mg/dl, and HDL-cholesterol 46.10 (SD=9.23)mg/dl.

Table 1 showed characteristics of pilot preconditions, whose data were presented as mean (SD) if the data distribution was normal and median (minimum – maximum) if the data distribution was not normal. CRM measured by the CMAQ questionnaire with a 1-5 scale, had a mean of 3.81 (SD=0.45), which represents high crew coordination, cockpit management, and personal capability.

**Table 1.** Characteristic of preconditions.

Preconditions	
**Adverse Mental State (Holmes Rahe Stress Scale)**	
Median (min - max)	88.0 (0-646)
**Adverse Physiological State (Fatigue Severity Scale)**	
Median (min - max)	3.44 (1 – 6.44)
**Physical/Mental Limitation (the difference of time to complete Trail Making Test A and B) -----seconds**	
Median (min - max)	14.58 (6.99 – 67.56)
**Personal Readiness (Job Content Questionnaire)**	
Psychological Demand----- Mean (SD)	2.31 (0.44)
Decision Latitude----- Mean (SD)	2.56 (0.49)
Skill Discretion----- Mean (SD)	3.02 (0.55)
Social Support----- Median (min - max)	3 (0-5)
**Cockpit Resource Management (Cockpit Management Assessment Questionnaire)**	
Mean (SD)	3.81 (0.45)

### Analysis

The correlations between the risk factors and preconditions are shown in Tables 2– 4. It includes the results of multivariate analysis for the correlation between internal and external risk factors with Adverse Mental State, Adverse Physiological State, Physical Mental Limitation, Cockpit Resource Management, Personal Readiness of Psychological Demand, Personal Readiness of Decision Latitude and Personal Readiness of Skill Discretion and Personal Readiness of Social Discretion.

**Table 2.** Correlation between internal and external risk factors and Adverse Mental Health.

Variable	ß	Standardized Error	95% Confidence Interval	p-value
**Adverse Mental Health**				
Constant	-68.33	49.915	-167,18 – 30,51	0.174
HDL-cholesterol	3.659	1.081	1.52 – 5.80	<0.001
*Burnout*				
Personal Burnout	1,.36	0.902	-0.45 – 3.12	0.141
Work related burnout	-0.482	0.959	-2.38 – 1.42	0.616
**Adverse Physiological State**				
Constant	2.233	0.787	0,673 – 3,793	0.005
Flight Time in one year	0.000	0.000	0.000 – 0.001	0.420
Flight Time in seven days	0.008	0.010	-0.012 – 0.028	0.427
Sleep duration	-0.137	0.079	-0.294 – 0.021	0.088
HDL-cholesterol	0.005	0.010	-0.014 – 0.024	0.583
*Burnout*				
Personal Burnout	0.021	0.008	0.005 – 0.037	0.009
Work Related Burnout	0.026	0.009	0.009 – 0.042	0.003
**Physical Mental Limitation**				
Constant	43.737	10.952	22,048 – 65,425	<0.001
Number of Sectors	1.512	0.752	0.022 – 3.001	0.047
Alcohol Drinking Habit	-3.181	1.979	- 7.10 – 0.739	0.111
Fasting Blood Glucose	-0.275	0.103	-0.479 – (-0.071)	0.009

Table 2 showed a significant correlation between HDL-cholesterol with Adverse Mental State (95% Confidence Interval/CI=1.52–5.80), and Personal (95%CI=0.005–0.037) and Work Related (95%CI=0.009–0.042) dimensions of Burnout with Adverse Physiological State. In addition, Table 2 also showed a significant correlation between the Number of Sectors in 24 hours (95%CI=0.022–3.001) and Fasting Blood Glucose (95%CI=(-0.479)–(-0.071)) with Physical Mental Limitation.

In conjunction with this, Table 3 also showed a significant correlation between Flight Time of one year (95%CI=0.000–0.001), Sleep Duration (95%CI=(-0.137)–(-0.013)), and Waist Circumference (95%CI =(-0.014)–(-0.002)) as factors contributing to the Psychological Demand dimension of Personal Readiness. In addition, Table 4 showed a significant correlation between Sleep Duration (95%CI=0.018–0.207) and client-related burnout (95%CI =(-0.011)–(-0.002)) as factors for the Social Support dimension of Personal Readiness.

**Table 3.** Correlation between internal and external factors and Personal Readiness (Psychological Demand).

Variable	ß	Standardized Error	95% Confidence Interval	p-value
Constant	1.813	0.603	0,617 – 3,008	0.003
Age	-0.003	0.004	-0.011 – 0.004	0.380
Flight time in 1 year	0.000	0.000	0.000 – 0.001	0.003
Flight time in 24 hours	-0.027	0.051	-0.128 – 0.073	0.592
Flight duty period	0.049	0.049	-0.048 – 0.147	0.319
Sleep duration	-0075	0.031	-0.137 – (-0.013)	0.019
Waist circumference	-0.008	0.003	-0.014 – (-0.002)	0.008
Systolic blood pressure	0.010	0.005	0.000 – 0.020	0.055
Diastolic blood pressure	0.001	0.009	-0.017 – 0.018	0.921
Triglyceride	0.000	0.001	-0.001 – 0.001	0.705
Burnout				
Personal Burnout	0.005	0.003	-0.001– 0.011	0.121
Work Related Burnout	0.002	0.003	-0.004 – 0.009	0.504

**Table 4.** Correlation between internal and external factors and Personal Readiness (Social Support).

Variable	ß	Standardized Error	95% Confidence Interval	p-value
Constant	2.544	0.435	1.683 – 3.405	<0.001
Flight Time in one year	0.000	0.000	-0.001 – 0.000	0.409
Alcohol Drinking Habit	0.125	0.076	-0.026 – 0.276	0.103
Sleep Duration	0.113	0.048	0.018 – 0.207	0.020
Triglyceride	0.001	0.001	-0.001 – 0.003	0.379
Burnout				
Personal Burnout	-0.008	0.005	-0.018 – 0.002	0.099
Work Related Burnout	0.002	0.005	-0.008 – 0.012	0.728
Client Related Burnout	-0.007	0.002	-0.011 – (-0.002)	0.004

## Discussion

This positive correlation showed that HDL cholesterol was not protective against Adverse Mental States. Nevertheless, these results should be cautiously interpreted. Several conditions may support this result. First, the subjects had a median age of 33 years with a mean HDL-cholesterol of 44.10 mg/dl, and by categorizing the HDL-cholesterol based on the guidance recommended by the Association of Endocrinologists in Indonesia (PERKENI), 30.3% of the subjects had low HDL-cholesterol (<40 mg/dl), 64.8% of the subjects had normal HDL-cholesterol (40-59 mg/dl), and 4.9% had high HDL-cholesterol (>60 mg/dl). These facts show that most of the subjects had normal HDL cholesterol levels. Second, the subjects were recruited at the Aviation Medical Center, a government health facility where pilots had their routine medical examination every six months. Generally, the participants came from communities that tended to have better health consciousness and may have had better health preparedness before taking the health examination. Third, the Adverse Mental Health was measured subjectively by self-reporting questionnaires (Holmes Rahe Stress Scale) that quantified stressors that happened in the last year, while the HDL-cholesterol levels were affected by the hypothalamic pituitary adrenal (HPA) axis that is dynamically influenced by the circadian rhythm and ultradian oscillation. ^34^ Lastly, the design of this study was cross-sectional, which meant that the correlation of variables could not determine causality.

It must also be noted that the correlation between HDL cholesterol and mental condition, which has already been analyzed in several studies, showed an inconsistent result. ^35, 36^ Aijanseppa et al. (2002) and Rice et al. (2010) showed no correlation between HDL cholesterol and depression, ^36^ while Ancelin et al. (2010) and Kim et al. (2004) showed a correlation between low HDL cholesterol and depression. On the other hand, Olusi and Fido (1996) and Shin at al. (2016) showed a correlation between higher HDL-cholesterol and depression. ^35^ The finding that was also verified by Kim et al. (2016), which showed a correlation between HDL-cholesterol and potential risk factors in mood disorder. ^37^ As a consequence, it would be beneficial to take into account the patients’ cholesterol level, as both mental health and cardiovascular system play an important role in their overall health. ^35^ Although the results from these studies may be controversial, the potential mechanism involved in the explanations was that the serotonergic systems affecting psychiatric disorders, such as serotonin, may affect psychologically driven behaviors, including mood changes, obsessive compulsive disorder, impulsivity, aggression, and suicide. Furthermore, low HDL cholesterol is correlated with low microviscosity of the brain membrane, and as a result, impaired serotonin transmission affects the concentration of serotonin and may increase the risk of mood changes. ^38^

This study also showed that the preconditions of pilots are affected by multiple factors, including internal and external factors. In terms of internal factors, this study showed that burnout, waist circumference, and fasting blood glucose levels correlated significantly with preconditions. Waist circumference and fasting blood glucose levels are known risk factors for metabolic syndrome and cardiovascular diseases. Personal and work-related burnout correlated significantly with adverse physiological states, which were measured using the fatigue severity scale. In addition, client-related burnout was significantly correlated with personal readiness in the social support dimension. In terms of external factors, Flight Time in one year, Number of Sectors in 24 h, and Sleep Period correlated significantly with preconditions. The number of sectors proved to be significantly correlated with physical and mental limitations. Flight time in one year and sleep period were significantly correlated with Personal Readiness in the dimension of psychological demand.

Workload for pilots, including flight time, duty time, and number of sectors, contributed not only to fatigue but also to mental fatigue or burnout. Cumulative workload chronically affected preconditions, which were classified into physical mental limitations and personal readiness. Both preconditions represent cognitive functions including memory, language, reasoning, problem solving, and decision-making. Nonetheless, the chronic effect of fatigue in the subjects could be identified by waist circumference, which is also a reliable indicator of abdominal obesity and fasting blood glucose. ^30, 39, 40^ Waist circumference and fasting blood glucose are two out of five parameters of Metabolic Syndrome. ^41^ Whitmer RA et al. (2008) found that central obesity increased the risk for dementia, while similar findings were demonstrated by Cho et al. (2019), who stated that older adults with normal weight but abdominal obesity had a higher risk of dementia than those without abdominal obesity. The pathophysiological mechanism between central obesity and blood sugar consists of several explanations. ^42^ Obesity is a chronic persistent inflammation that induces the secretion of a proinflammatory condition that also happens in the brain and causes neurodegeneration. Simultaneously, excess proinflammatory conditions lead to insulin resistance and, hence, metabolic syndrome, which is correlated with low cognitive function. Additionally, persistent inflammation leads to leptin resistance, reduced lipocalin secretion, and induces further neurodegeneration that could progress to neurodegenerative disorders. Finally, obesity could compromise the blood-brain barrier and cause cerebrovascular dysfunction and cognitive impairment. ^38, 40^

The number of sectors proved to be significantly correlated with physical and mental limitations. The number of sectors represented the frequency of takeoff and landing, which are the most intense phases of flights, and cause high workload, which considerably impacts pilots’ performance. Pilots who fly in multiple sectors cumulatively lead to higher flight times or flight duty times in the last 24 hours. Moreover, when an excessive workload occurs for one year, ultimately increasing the flight time in one year, which in this research was significantly correlated with the personal readiness dimension of psychological demand. Pilots are highly competent human resources in aviation, and their cognitive functions are expected to be ready psychologically and physically under all circumstances. The personal readiness of pilots can be attributed to sleep restrictions. The rest period for pilots is regulated in accordance with the Ministry Decree, but it might not be efficiently carried out when it is not properly managed to its restorative value. The most efficient restorative value can be achieved with sufficient sleep. This study showed that sleep duration was correlated with personal readiness in the dimensions of psychological demand and social support.

Although this study did not show a correlation between the risk factors of CRM and Personal Readiness in the dimension of Skill Discretion and Decision Latitude, it did not disregard the fact that it is beneficial to pay attention to the internal and external factors that affect CRM and personal readiness, specifically in the dimension of Skill Discretion and Decision Latitude. Remarkable cognitive function is required for pilots to maintain good CRM and Personal Readiness, especially in the dimensions. In fact, in the bivariate analysis, this study found a correlation between personal and work-related burnout and CRM. Pertaining to the decision latitude and skill discrepancy dimensions of Personal Readiness, this study found a correlation between sleep duration, alcohol drinking habits, fasting blood glucose, and burnout. In addition, several accident and serious incident investigation reports showed the importance of CRM and Personal Readiness. One of the accident investigation reports in Indonesia that highlighted compromised CRM and Personal Readiness was report number KNKT.11.05.10.04 on May 7, 2011. The National Transport Safety Committee declared that the contributing factors in these accidents can be attributed to the lack of professional CRM, including steep authority, communication, and checklist-reading. In addition, compromised Personal Readiness was also stated as the experience of the crew on this particular aircraft was minimal, both pilots had inadequate training, and the PIC experienced a competency regression from his last type of rating. ^15^

This study had several benefits. First, this is the first HFACS precondition study among pilots in Indonesia. Second, the focus of this study was on the five preconditions relating to pilot performance. Third, the risk factors consisted of both external and internal factors. Nevertheless, there were also limitations because some of the variables were measured subjectively using a self-reporting questionnaire. Therefore, it will be beneficial to conduct future research in a larger sample cohort study design to increase the reliability and representation of both independent and dependent variables. Despite the benefits, there is one limitation that must be taken into consideration, as the study only measured sleep duration and not sleep quality. Sleep quality is a crucial factor for fatigue management. Therefore, future research should consider sleep quality.

## Ethical considerations

The study was approved by the Medical Research Ethics Committee, Faculty of Medicine, University of Indonesia (certificate number KET1139/UN2). F1/ETIK/PPM.00.02/2024, dated 5 August, 2024.

The participants provided written informed consent to participate in the study. The data were collected by filling in anonymous self-reporting questionnaires and performing the Trail Making Test A and B. The written informed consent were obtained by giving a thorough explanation of the procedure and ethic of the study, suggesting each participant to read the explanation and ask questions related to their participation. Laboratory test results, including plasma lipid and glucose levels, and physical examination results (height, weight, waist circumference, and blood pressure) were collected from the participants’ medical records.

## Data Availability

Figshare: Internal and external risk factors analysis on pilot precondition in Indonesia. https://doi.org/10.6084/m9.figshare.28794371 ^43^ The project contains the following underlying data:
• Pilots_preconditions.xlsx. The data are available under the terms of the Creative Commons Attribution 4.0 International license (CC-BY 4.0).
