# Supplementary material for: Internal and external risk factors analysis on pilot precondition in Indonesia
Source: F1000Res. 2025 Oct 20;14:458. Originally published 2025 Apr 24. [Version 4] doi: 10.12688/f1000research.163883.4 (PMC12219539; doi:10.12688/f1000research.163883.4)
Supplement: Peer review history

# Reviewer report: Reviewer response for version 4

**DOI:** 10.5256/f1000research.189699.r425496

**Published:** 2025-11-22

**Authors:** Jirat Bhanpato

**Affiliations:** 1 Aerospace Engineering, Georgia Institute of Technology, Atlanta, Georgia, USA

Thank you for the revisions. No further comments.

Is the work clearly and accurately presented and does it cite the current literature?

Yes

If applicable, is the statistical analysis and its interpretation appropriate?

I cannot comment. A qualified statistician is required.

Are all the source data underlying the results available to ensure full reproducibility?

Partly

Is the study design appropriate and is the work technically sound?

Yes

Are the conclusions drawn adequately supported by the results?

Partly

Are sufficient details of methods and analysis provided to allow replication by others?

Yes

Reviewer Expertise:

aviation safety, aviation environmental impacts, airline operations, aerospace systems engineering, machine learning

I confirm that I have read this submission and believe that I have an appropriate level of expertise to confirm that it is of an acceptable scientific standard.

# Reviewer report: Reviewer response for version 3

**DOI:** 10.5256/f1000research.186350.r406698

**Published:** 2025-10-11

**Authors:** Jirat Bhanpato

**Affiliations:** 1 Aerospace Engineering, Georgia Institute of Technology, Atlanta, Georgia, USA

This paper provides a study of correlations between internal and external risk factors and pilot preconditions. The authors describe the background of the problem by discussing the significance of human factors in aviation accidents and incidents. The various layers of HFACS are discussed, with emphasis on the preconditions of unsafe acts, which is modeled as the layer prior to human errors, thereby motivating the importance of the study’s focus. The methodology is described in detail, including data collection methods, participants pool, the consent of the participants, as well as ethics approval necessary to collect data. The measurements of independent and dependent variables are discussed sufficiently.

 Overall, the study provides valuable contributions to aviation safety and support further improvements to fatigue risk management and safety management system, especially in Indonesia.

 In future study, it would be interesting to see data collected over a longer time span since certain factors, such as pilot schedule, may vary with seasonality in operational patterns.

**Minor feedback for Background section:**

 Capitalization of the various layers of HFACS is inconsistent *(“The Third layer”, “The second layer”, “The last or first layer”*). 

 In the statement *“The Cockpit Resource Management (CRM) is described as one or more of these conditions including lack of communication, power distance/authority, lack of teamwork, lack of pre-flight briefing, lack of leadership”,* the authors refer to CRM as negative conditions. However, CRM is a tool meant to reduce such conditions. Recommend rewording to *“Ineffective Cockpit Resource Management (CRM) is described as one or more of these conditions including lack of…” *or similar.

 Recommend rewording “ *Lastly, the precondition of unsafe acts is Personal Readiness”* to *“The last precondition of unsafe acts is Personal Readiness”*.

 The authors cited the NTSC database (Ref # 18) that “ *human factors accounted for 66 out of 69 accidents and serious incident investigation reports from 2014 to 2023 in Indonesia”.* However, this reference is missing citation text. 

**Feedback for Results section:**

 Table 2 shows correlations of multiple preconditions (Adverse Mental State, Adverse Physiological State, Physical Mental Limitation) while the caption only refers to Adverse Mental State.

 The author states that Tables 2-4 include correlation results for dependent variables: “ *Adverse Mental State, Adverse Physiological State, Physical Mental Limitation, Cockpit Resource Management, Personal Readiness of Psychological Demand, Personal Readiness of Decision Latitude and Personal Readiness of Skill Discretion and Personal Readiness of Social Discretion”*. However, results for *Cockpit Resource Management, Personal Readiness of Decision Latitude, Personal Readiness of Skill Discretion, and Personal Readiness of Social Discretion* are not shown.

 Furthermore, the term “Personal Readiness of Social Discretion” is first introduced here. The lists of independent variables being evaluated are also not consistent across the dependent preconditions. 

 If these tables omit certain statistical analyses, the criteria which were used to do so should be stated.

**Minor feedback for Discussion section:**

 The statement *“Moreover, when an excessive workload occurs for one year, ultimately increasing the flight time in one year, which in this research was significantly correlated with the personal readiness dimension of psychological demand” *is an incomplete sentence. 

 The acronym PIC (Pilot-in-Command?) is not defined.

Is the work clearly and accurately presented and does it cite the current literature?

Yes

If applicable, is the statistical analysis and its interpretation appropriate?

I cannot comment. A qualified statistician is required.

Are all the source data underlying the results available to ensure full reproducibility?

Partly

Is the study design appropriate and is the work technically sound?

Yes

Are the conclusions drawn adequately supported by the results?

Partly

Are sufficient details of methods and analysis provided to allow replication by others?

Yes

Reviewer Expertise:

aviation safety, aviation environmental impacts, airline operations, aerospace systems engineering, machine learning

I confirm that I have read this submission and believe that I have an appropriate level of expertise to confirm that it is of an acceptable scientific standard, however I have significant reservations, as outlined above.

# Response

**Published:** 2025-10-14

**Authors:** Inne Yuliawati

**Affiliations:** Medical Science,Doctoral Study, Universitas Indonesia, Jakarta Pusat, Indonesia

Thank you for the thorough report. Please find the revision below:

 1. Background:

- explanation: The CRM in *“The Cockpit Resource Management (CRM) is described as one or more of these conditions including lack of communication, power distance/authority, lack of teamwork, lack of pre-flight briefing, lack of leadership”… *the authors refer the CRM as one of the classification in the HFACS Level 2 The Preconditions of Unsafe Acts, thus the classification itself includes the  lack of communication, power distance/authority, lack of teamwork, lack of pre-flight briefing, lack of leadership. The paragraphs above and below CRM also describes the classifications in the HFACS Level 2.
- The reference no.18 is https://knkt.go.id/investigasi

2. Results:

- I will change explanation of paragraph 3 into: The correlations between the risk factors and preconditions are shown in Tables 2– 4. It includes the results of multivariate analysis for the correlation between internal and external risk factors with Adverse Mental State, Personal Readiness dimension of psychological demand and Personal Readiness dimension of Social Support.
- And also revised the social discretion to “social support”

3. Discussion

- I'll rewrite the sentence of *“Moreover, when an excessive workload occurs for one year, ultimately increasing the flight time in one year, which in this research was significantly correlated with the personal readiness dimension of psychological demand” * starting from the beginning of the paragraph, to: “The Number of Sectors proved to be significantly correlated with Physical and Mental Limitations which represented the cognitive function. The Number of Sectors are the frequency of take-offs and landings, as the most intense phases of flights that considerably impacts pilots’ performance. Pilots who fly multiple sectors, extended flight time or duty time, cumulatively experience excessive workload that could compromise their cognitive function. This study showed that Flight Time in One Year was significantly correlated with the Personal Readiness dimension of Psychological Demand. Pilots are highly competent human resources in aviation, and their cognitive functions are expected to be ready psychologically and physically under all circumstances. Other than that, the personal readiness of pilots can be attributed to sleep restrictions. The rest period for pilots is regulated in accordance with the Ministry Decree, but it might not be efficiently carried out when it is not properly managed to its restorative value. The most efficient restorative value can be achieved with sufficient sleep. This study showed that sleep duration was correlated with personal readiness in the dimensions of psychological demand and social support.”
- Add The acronym of PIC: Pilot-in-Command

# Reviewer report: Reviewer response for version 3

**DOI:** 10.5256/f1000research.186350.r406435

**Published:** 2025-08-19

**Authors:** Zbigniew Wochyński

**Affiliations:** 1 Polish Air Force University, Deblin, Poland

The article, after corrections, is very good.

 Sincerely,

 Zbigniew Wochyński

Is the work clearly and accurately presented and does it cite the current literature?

Yes

If applicable, is the statistical analysis and its interpretation appropriate?

Yes

Are all the source data underlying the results available to ensure full reproducibility?

Yes

Is the study design appropriate and is the work technically sound?

Yes

Are the conclusions drawn adequately supported by the results?

Yes

Are sufficient details of methods and analysis provided to allow replication by others?

Yes

Reviewer Expertise:

aviation environment

I confirm that I have read this submission and believe that I have an appropriate level of expertise to confirm that it is of an acceptable scientific standard.

# Reviewer report: Reviewer response for version 2

**DOI:** 10.5256/f1000research.183469.r391630

**Published:** 2025-06-26

**Authors:** Zbigniew Wochyński

**Affiliations:** 1 Polish Air Force University, Deblin, Poland

Reviewer report

 The scientific work pl. "Internal and external risk factors analysis on pilot precondition in Indonesia" is very important from the point of view of flight safety. It is helpful for people managing aviation safety. The work is of a research nature and meets the conditions for indexing in F1000 Research.

 In their work, the authors paid special attention to the human factor, which is the cause of air accidents in Indonesia (66 accidents out of 69), this is a strong justification for the purpose of their scientific work. In the research, they took into account internal factors: cholesterol-HDL, glucose, triglycerols, waist circumference, mental fatigue and alcohol consumption habits. As well as external factors related to work: flight time, time on flight duties, number of sectors, unplanned flight duties and sleep time. These factors have diagnostic value and can undoubtedly be the cause of air accidents. The authors used selective blood biochemical indicators in the work, which can partially indicate disturbing episodes in the pilot's body, e.g.: long exposure to a poor diet, alcohol drinking and a long period of physical inactivity. It is a pity that the authors did not use additional indicators with extended diagnostic values, e.g. bioelements and some proteins. The reviewer hopes that in future work the authors of the study should use lipid indicators of high diagnostic value, which could show the physical fitness and proper nutrition of pilots. This could constitute a recommendation (conclusions) for pilots regarding physical activity and diet as a mental hygiene procedure (reducing stress). A well-nourished pilot has a low need for alcohol drinking, has no sleep disorders and regenerates quickly. Without physical activity and diet, the pilot will not achieve the optimal psychomotor level necessary in the conditions of his work. With the help of these two factors, the level of cognition increases, brain capacity increases, the lipid profile normalizes. In depression, this is a basic condition for treatment. In depression, the concentration of tryptophan decreases. Tryptophan is an essential exogenous amino acid, which serves, among other things, as a substrate for the production of neuroactive substances. One of them is serotonin (5-hydroxytryptamine), which is an important neurotransmitter involved in the control of processes in the central nervous system, directly related to mood, anxiety and cognitive processes.

Comments and suggestions

 1. Section "Methods"

 Write the same content in the following structure:

 Subject:

 Measurement:

 Procedure and design:

 Ethics:

 Statistical analysis:

 2. Section "Results"

 The first sentence should be placed in the section "Methods"

 3. Section "Discussion"

Suggestion for Authors

 The reviewer proposes to cite 3 scientific papers in the final part of the paper:

 Our studies provide a good basis for future studies on pilots, taking into account factors such as: diet quality, physical activity, and sleep quality, monitoring them using lipid indicators with high diagnostic value in terms of physical performance and health (Ref 1,2,3). This direction of research will certainly contribute to maintaining a balance between work and body regeneration in pilots, which may be useful from the point of view of flight planning.

 1.Wochyński Z. 2025 (Ref 1)

 2.Wochyński Z, et al. 2020 (Ref 2)

 3.Wochyński Z., et al. 2016 (Ref 3)

 Yours sincerely

 Reviewer

Is the work clearly and accurately presented and does it cite the current literature?

Yes

If applicable, is the statistical analysis and its interpretation appropriate?

Yes

Are all the source data underlying the results available to ensure full reproducibility?

Yes

Is the study design appropriate and is the work technically sound?

Yes

Are the conclusions drawn adequately supported by the results?

Yes

Are sufficient details of methods and analysis provided to allow replication by others?

Yes

Reviewer Expertise:

aviation environment

I confirm that I have read this submission and believe that I have an appropriate level of expertise to confirm that it is of an acceptable scientific standard.

## References

[1] : Evaluation of selected elements of fitness and physiological diagnostics of military pilots as a factor in flights safety. *Frontiers in Physiology* .2025;16: 10.3389/fphys.2025.1558786 10.3389/fphys.2025.1558786 PMC1208271340386623

[2] : The assessment of the impact of a training process on the habituation of the vestibular-vegetative system, using a special rotational test as a condition of maintaining flight safety. *International Journal of Occupational Medicine and Environmental Health* .2020;33(4) : 10.13075/ijomeh.1896.01515 497-506 10.13075/ijomeh.1896.01515 32519680

[3] : Effect of centrifuge test on blood serum lipids index of cadet pilots. *Annals of Agricultural and Environmental Medicine* .2015;23(1) : 10.5604/12321966.1184693 1-5 10.5604/12321966.1184693 27007513

# Reviewer report: Reviewer response for version 2

**DOI:** 10.5256/f1000research.183469.r391284

**Published:** 2025-06-26

**Authors:** Xinyu Zhao

**Affiliations:** 1 Arizona State University, Tempe, Arizona, USA

Revisions are good. No more comments.

Is the work clearly and accurately presented and does it cite the current literature?

Partly

If applicable, is the statistical analysis and its interpretation appropriate?

Yes

Are all the source data underlying the results available to ensure full reproducibility?

Partly

Is the study design appropriate and is the work technically sound?

Yes

Are the conclusions drawn adequately supported by the results?

Yes

Are sufficient details of methods and analysis provided to allow replication by others?

Yes

Reviewer Expertise:

machine learning

I confirm that I have read this submission and believe that I have an appropriate level of expertise to confirm that it is of an acceptable scientific standard.

# Reviewer report: Reviewer response for version 2

**DOI:** 10.5256/f1000research.183469.r391283

**Published:** 2025-06-16

**Authors:** Neelakshi Majumdar

**Affiliations:** 1 Mechanical Engineering, University of Arkansas System, Fayetteville, Arkansas, USA

The authors have made appropriate updates to the paper based on the review comments.

Is the work clearly and accurately presented and does it cite the current literature?

Partly

If applicable, is the statistical analysis and its interpretation appropriate?

I cannot comment. A qualified statistician is required.

Are all the source data underlying the results available to ensure full reproducibility?

Partly

Is the study design appropriate and is the work technically sound?

Yes

Are the conclusions drawn adequately supported by the results?

Partly

Are sufficient details of methods and analysis provided to allow replication by others?

Yes

Reviewer Expertise:

Aerospace systems engineering, aerospace safety, transportation and systems safety, human factors, next-generation transportation, aviation operations, accident modeling, data analysis, machine learning, natural language processing

I confirm that I have read this submission and believe that I have an appropriate level of expertise to confirm that it is of an acceptable scientific standard.

# Reviewer report: Reviewer response for version 1

**DOI:** 10.5256/f1000research.180302.r385545

**Published:** 2025-06-06

**Authors:** Xinyu Zhao

**Affiliations:** 1 Arizona State University, Tempe, Arizona, USA

**Strengthen Abstract**:

- Clearly describe the study's motivation and contributions.
- Remove incomplete sentences

**Justify unexpected correlations**:

- The HDL-Adverse Mental State result needs stronger interpretation or a note acknowledging its speculative nature.

**Correct formatting and reporting errors**:

- Standardize decimal points, CI notation, and remove arbitrary capitalizations.

**Clarify data availability**:

- Describe dataset columns in the manuscript and specify which were used for each analysis.

Is the work clearly and accurately presented and does it cite the current literature?

Partly

If applicable, is the statistical analysis and its interpretation appropriate?

Yes

Are all the source data underlying the results available to ensure full reproducibility?

Partly

Is the study design appropriate and is the work technically sound?

Yes

Are the conclusions drawn adequately supported by the results?

Yes

Are sufficient details of methods and analysis provided to allow replication by others?

Yes

Reviewer Expertise:

machine learning

I confirm that I have read this submission and believe that I have an appropriate level of expertise to confirm that it is of an acceptable scientific standard, however I have significant reservations, as outlined above.

# Reviewer report: Reviewer response for version 1

**DOI:** 10.5256/f1000research.180302.r381090

**Published:** 2025-05-23

**Authors:** Neelakshi Majumdar

**Affiliations:** 1 Mechanical Engineering, University of Arkansas System, Fayetteville, Arkansas, USA

The authors conducted an interesting study on aviation accidents and incidents.

 The paper has grammatical and sentence formation mistakes, e.g., first sentence in the Background section. Also, unnecessary capitalization of variable names or causes categories throughout the paper, e.g., “Age, Burnout,” etc. Same in “Results” section and throughout the paper.

 Authors are encouraged to proofread the paper to make it competent.

 The background of the abstract should be revised to include the “background” of the research topic. What is this study about, motivation and existing work is missing.

 Incomplete sentence: “The independent variables included internal (Age...) and external (...).”

 The paragraph starting with “Ultimately, Aviation safety, specifically pilot errors and preconditions, are a major concern in Indonesia.” Should be in the beginning in the background section. First state the problem, then mention the methodologies like the HFACS.

 Reference missing for the statement “Additionally, the NTSC recorded that human factors accounted for 66 out of 69 accidents and serious incident investigation reports from 2014 to 2023 in Indonesia.”

 Some examples for the survey questions will help reader get a deeper context of the study.

 The results section lacks clear subheadings or structured organization (e.g., demographic characteristics, physiological results, analysis outcomes), which makes it difficult to follow.

 Phrases like “subjects were collected” are vague and ethically problematic.

 There are inconsistencies and errors in the reporting of statistical values. For example, some confidence intervals are reported with incorrect punctuation (e.g., commas instead of periods), and some variables are referenced without clear indication of the statistical test used.

 The connection between risk factors and preconditions (e.g., HDL-cholesterol and Adverse Mental State) is not sufficiently explained in the context of existing literature in the results section.

 Terms like “Decision Latitude,” and “Skill Discretion” are introduced without clear definitions in the results.

Is the work clearly and accurately presented and does it cite the current literature?

Partly

If applicable, is the statistical analysis and its interpretation appropriate?

I cannot comment. A qualified statistician is required.

Are all the source data underlying the results available to ensure full reproducibility?

Partly

Is the study design appropriate and is the work technically sound?

Yes

Are the conclusions drawn adequately supported by the results?

Partly

Are sufficient details of methods and analysis provided to allow replication by others?

Yes

Reviewer Expertise:

Aerospace systems engineering, aerospace safety, transportation and systems safety, human factors, next-generation transportation, aviation operations, accident modeling, data analysis, machine learning, natural language processing

I confirm that I have read this submission and believe that I have an appropriate level of expertise to confirm that it is of an acceptable scientific standard, however I have significant reservations, as outlined above.

# Response

**Published:** 2025-06-02

**Authors:** Inne Yuliawati

**Affiliations:** Medical Science,Doctoral Study, Universitas Indonesia, Jakarta Pusat, Indonesia

Thank you for the thorough report. I will revise the writing but It will be best to do it after the reports are completed from other reviewers.

 Please find the revision below that I will put after the review:

1. I will capitalize each Variable name in the whole section
2. Background:
3. Pilot errors and preconditions, are a major concern in Indonesia. The Human Factors Analysis and Classification System recognizes the preconditions of pilots as the imminent layer prior to errors and multiple risk factors affected pilots’ precondition physiologically, psychologically and psychosocially. This study aimed to analyze the correlation between the internal and external risk factors, and pilot preconditions in Indonesia.
4. The independent variables divided into internal factors consisted of Age, Burnout, Metabolic Syndrome parameters; and external factors consisted of Flight Time, Duty Time, Unscheduled Flight Duty, Number of Sectors, Sleep Duration.
5. Add the sentence of “Pilot errors and preconditions, are a major concern in Indonesia” to the beginning of background, as suggested
6. Add reference of the NTSC database: https://knkt.go.id/investigasi as number 18
7. change section: Characteristic to “demographic characteristics’ and analysis to “analysis outcomes”
8. Revise the commas into period in the confidence interval
9. Add more explanation on the correlation between HDL-cholesterol and adverse mental state, as the third paragraph of Discussion:

“Previous researches showed that the correlation of HDL-cholesterol and Adverse Mental State was diverse that it requires a deep understanding and discernment to respond on the result. Nasrallah et al. (2018) in the journal Current Psychiatry which concluded various studies on the relationship between cholesterol and mental disorders stated that the relationship is complex and confusing. This phenomena explaines that the brain, which only weighs 2% of the body's weight, contains 25% of the body's cholesterol, and cholesterol plays an important role in the function of transmission neurons. The roles of cholesterol in the brain includes (1) produce neuroactive steroids (NASs) that was synthesized from cholesterol;  (2) modulate brain processes; (3) interact g-aminobutyric acid, N-methyl-d-aspartate, serotonin receptors and neurotrophins such as nerve growth factors. NASs play a role in mood and cognitive regulation and regulate synapse plasticity, apoptosis and neuroprotection. In order for the brain to function properly, neuroactive steroids must be maintained at normal levels, because low levels can cause depression, inflammation of the nerves, epilepsy, multiple sclerosis and psychosis. However, high levels can cause attention deficits or hyperactivity disorders and stress. Thus, NASs such as pregnane, androstane and sulfated neurosteroids that were synthesized from cholesterol are important molecules for neuropsychiatric activity. Cholesterol is stated as a versatile molecule which is a major component of neuronal cell membranes and a precursor of many signaling molecules. Thus, Nasrallah et al. strongly suggested that it is crucial to protect the brain functions by managing the adverse mental state with both cholesterol and cardiovascular therapeutic management in sync. [add reff: Nasrallah HA. The puzzling relationship between cholesterol and  psychopathology. Current Psychiatry. 2018;(17)1]

 10. Add more information on decision latitude and skill discretion in the method, at the paragraph of methods, section procedure:

 Personal Readiness was measured by the Job Content Questionnaire (JCQ). ^33^ JCQ measures four dimensions of personal readiness, with a total of 34 questions. Each dimension followed by question amount was psychological demand with nine questions, decision latitude with 12 questions, skill discretion with five questions, and social support at work with eight questions. The answers to each question were measured with a Likert scale ranging from 1 to 4, where the higher the value, the more the participant agrees with each question on the questionnaire. “ The assessment is based on the average value of each dimensions. For Psychological Demand dimension, higher average value indicates a higher psychological tension that was perceived in carrying out the work. Meanwhile, higher average value for the Decision Latitude dimensions indicates the subject perceived lower participation at the individual level and at the organizational level. Then, the higher average value for Social Support dimension shows higher support from superiors and coworkers in carrying out the work. Finally, the higher average value for the Skill Discretion dimension shows higher flexibility in creativity and ability to carry out the work.”

 The final data from the questionnaire included the average scores obtained for each dimension. The questionnaire has a reliability value of Cronbach Alpha 0.71-0.86 (DOI: 10.1037//1076-8998.3.4.322).

# Response

**Published:** 2025-06-09

**Authors:** Inne Yuliawati

**Affiliations:** Medical Science,Doctoral Study, Universitas Indonesia, Jakarta Pusat, Indonesia

Thank you for the thorough report. Please find the revision below:

 1. Abstract, Background: Pilot errors and preconditions, are a major concern in Indonesia. The Human Factors Analysis and Classification System recognizes the preconditions of pilots as the imminent layer prior to errors and multiple risk factors affected pilots’ precondition physiologically, psychologically and psychosocially. This study aimed to analyze the correlation between the internal and external risk factors, and pilot preconditions in Indonesia.

 2. Correlation of HDL-cholesterol and AMS: add more explanation on the correlation between HDL-cholesterol and adverse mental state, as the third paragraph of Discussion: “Previous researches showed that the correlation of HDL-cholesterol and Adverse Mental State was diverse that it requires a deep understanding and discernment to respond on the result. Nasrallah et al. (2018) in the journal Current Psychiatry which concluded various studies on the relationship between cholesterol and mental disorders stated that the relationship is complex and confusing. This phenomena explaines that the brain, which only weighs 2% of the body's weight, contains 25% of the body's cholesterol, and cholesterol plays an important role in the function of transmission neurons. The roles of cholesterol in the brain includes (1) produce neuroactive steroids (NASs) that was synthesized from cholesterol;  (2) modulate brain processes; (3) interact g-aminobutyric acid, N-methyl-d-aspartate, serotonin receptors and neurotrophins such as nerve growth factors. NASs play a role in mood and cognitive regulation and regulate synapse plasticity, apoptosis and neuroprotection. In order for the brain to function properly, neuroactive steroids must be maintained at normal levels, because low levels can cause depression, inflammation of the nerves, epilepsy, multiple sclerosis and psychosis. However, high levels can cause attention deficits or hyperactivity disorders and stress. Thus, NASs such as pregnane, androstane and sulfated neurosteroids that were synthesized from cholesterol are important molecules for neuropsychiatric activity. Cholesterol is stated as a versatile molecule which is a major component of neuronal cell membranes and a precursor of many signaling molecules. Thus, Nasrallah et al. strongly suggested that it is crucial to protect the brain functions by managing the adverse mental state with both cholesterol and cardiovascular therapeutic management in sync. [add reff: Nasrallah HA. The puzzling relationship between cholesterol and  psychopathology. Current Psychiatry. 2018;(17)1]

 3. Formatting errors of CI: Revise the commas into period in the confidence interval (all table)

 4. Describe dataset columns in the manuscript and specify which were used for each analysis (all tables)
